# Attenuation of Human Growth Hormone-Induced Rash With Graded Dose Challenge

**DOI:** 10.7759/cureus.27920

**Published:** 2022-08-12

**Authors:** Jake Mann, Dennis Caruana, Evelyn Luo, Eric Gottesman, Nidhi Agrawal, Daniel Lozeau, Justina Hessel, Melissa Neumann, Sameer Khanijo, Zubair Hasan, Khizer Rizvi, Regina Gunther, Daniel Donovan, Derek Chan, Mary Lee-Wong, Anthony M Szema

**Affiliations:** 1 Medicine, West Virginia School of Osteopathic Medicine, Lewisburg, USA; 2 Orthopedics, Yale University School of Medicine, New Haven, USA; 3 Division of Pharmacology Critical Care, North Shore University Hospital, Manhasset, USA; 4 ICU-Pulmonary, Critical Care and Sleep Medicine, North Shore University Hospital, Manhasset, USA; 5 Division of Endocrinology, Diabetes, and Metabolism, New York University Langone Medical Center, New York, USA; 6 Dermatology, Stony Brook University, Stony Brook, USA; 7 Division of Pulmonary and Critical Care, North Shore University Hospital, Manhasset, USA; 8 Medicine, North Shore University Hospital, Manhasset, USA; 9 Emergency Medicine, North Shore University Hospital, Manhasset, USA; 10 Medicine, Icahn School of Medicine at Mount Sinai, New York, USA; 11 Dermatology, All Dermis Dermatology, PC, New York, USA; 12 Allergy and Immunology, Mount Sinai Hospital, New York, USA; 13 Division of Allergy and Immunology, Division of Pulmonary and Critical Care, Donald and Barbara Zucker School of Medicine at Hofstra/Northwell, Manhasset, USA

**Keywords:** familial human growth hormone deficency, growth hormone replacement therapy, growth hormone therapy, growth hormone deficiency, growth hormone, desensitization, urticaria, atopic dermatitis, human growth hormone deficiency

## Abstract

Adult growth hormone (GH) deficiency is rare and requires replacement with extrinsic/synthetic injection. GH hypersensitivity has been reported; specifically, atopic patients may develop rashes from somatotropin therapy. Allergic and non-allergic skin reactions to recombinant human GH are uncommon and infrequently reported. We describe a graded-dose challenge with intravenous Norditropin® in a 65-year-old atopic adult woman who developed a severe whole-body rash with Norditropin FlexPro® administration on several occasions but was negative on skin-prick testing to Norditropin® percutaneously and intradermally, but the patch testing was positive for gold and nickel.

The patient was registered as a direct admission to the emergency room at a university hospital for a rapid antigen coronavirus disease 2019 (COVID-19) testing after having received two COVID-19 vaccinations and re-testing four months after vaccination. She was then directly admitted to a non-COVID-19 intensive care unit with direct bedside supervision by a registered nurse and a physician board certified in internal medicine, allergy/immunology, and pulmonary diseases. The patient brought a Norditropin® pen which our pharmacy team attached to a compatible syringe for dilutions. A graded dose challenge at a final dosage of 0.1 mL was performed and the patient was monitored for allergic and other adverse drug reactions, which did not occur. At the time of writing this case report, the patient has been maintained on Norditropin FlexPro® 0.1 mL and has not experienced any adverse reactions, including recurrent skin eruptions.

The case presented is the first to describe a patient who successfully tolerated a graded dose challenge of an adult patient to GH replacement therapy (as Norditropin®) under supervision in an intensive care unit, whereas prior to reporting of this case, a graded dose challenge to GH replacement therapy had only been successfully performed in a child using another formulation of somatotropin (Humatrope®). Hence, this case lends support that graded dose challenge with somatotropin analogs may be considered for patients with isolated GH deficiency such as in the case presented here.

## Introduction

Adult growth hormone deficiency (GH) also known as somatotropin is due to the inhibition of GH release from the anterior pituitary gland by a variety of mechanisms [1]. Adults need to have an adequate level of GH to maintain appropriate bone mineral density, body composition, cardiovascular health, and reproductive health [2]. In the human brain, the hypothalamus first releases growth hormone-releasing hormone (GHRH) through the hypophyseal portal system to signal the anterior pituitary gland to release somatotropin. Somatotropin is then released into the systemic circulation and induces insulin-like growth factor-1 (IGF-1) production in the liver [1]. Both GH and IGF-1 work by maintaining an appropriate level of gluconeogenesis and fat lipolysis to promote the overall growth of the body [2].

GH deficiency is caused by several insults, including, but not limited to, hypopituitarism, compression by an intrasellar mass, ischemia from radiation therapy, or an idiopathic mechanism [1]. Chemotherapy agents have also been shown to increase the risk of GH deficiency due to the generation of anti-cytotoxic T-lymphocyte-associated protein 4 antibodies to the pituitary gland causing hypopituitarism [3]. Reduced serum levels of IGF-1 and GH in combination with abnormal glucagon or insulin stimulation testing confirm the diagnosis of GH deficiency, which may be treated by subcutaneous injection of somatotropin analogs [4].

Adult GH deficiency is rare. Approximately 1:100,000 adult population and 1:10,000 children worldwide are diagnosed with adult GH deficiency annually [5,6]. Magnetic resonance imaging (MRI) scan of the pituitary gland can distinguish congenital versus acquired GH deficiency [7]. Congenital GH deficiency may occur concomitantly with deficiency of other pituitary hormones or result in an isolated loss of GH without deficiency of other pituitary hormones. Isolated GH deficiency is rare.

GH hypersensitivity has been reported; specifically, atopic patients may develop rashes from somatotropin therapy [8]. Allergic and non-allergic skin reactions to recombinant human growth hormone (rhGH) are uncommon and infrequently reported. Moreover, mechanisms are incompletely understood. In one case, skin reaction was not felt to be an exacerbation of atopic dermatitis [8]. An intravenous desensitization procedure with Humatrope® has been described in a nine-year-old boy who developed recombinant GH hypersensitivity [9]. Here, we describe a graded dose challenge with intravenous Norditropin® in an atopic adult woman who developed a severe whole-body rash with Norditropin FlexPro® administration on several occasions but was negative on skin-prick testing to Norditropin® both percutaneously and intradermally.

## Case presentation

A 65-year-old lady developed two episodes of a severe maculopapular erythematous rash on the thighs, torso, and inner arms after receiving Norditropin FlexPro®. Two years prior, the patient had presented with lassitude, lethargy, insomnia, and generalized weakness. An MRI scan of the pituitary was normal. A glucagon stimulation test was abnormal in that glucagon was unable to adequately stimulate the release of GH. A concurrent low level of GH at 6.7 ng/mL (reference range: ≤7.1 ng/mL) and low-normal baseline level of IGF-1 of 87 mg/mL (reference range: 41-273 mg/mL) confirmed the diagnosis of adult GH deficiency. The following lab values were within normal limits: adrenocorticotropic hormone (ACTH), prolactin, thyroid-stimulating hormone (TSH), free T4, follicle-stimulating hormone (FSH), and luteinizing hormone (LH) (Table 1). The patient’s antinuclear antibody titer was elevated (1:80). Although chronic urticaria is a clinical diagnosis, the <16% histamine release test was normal.

**Table 1:** Results of case presentation laboratory studies. ACTH = adrenocorticotropic hormone; FSH = follicle-stimulating hormone; TSH = thyroid-stimulating hormone; LH = luteinizing hormone

Laboratory study	Result	Reference range
ACTH, serum	6 pg/mL	6–50 pg/mL
TSH, serum	2.16 mIU/L	0.4–4.5 mIU/L
Free T4, serum	1.3 ng/dL	0.8–1.8 ng/dL
FSH, serum	75.8 mIU/mL	23–116 mIU/mL
LH, serum	37.8 mIU/L	10-54.7 mIU/mL
Prolactin, serum	8.1 ng/mL	<25 ng/mL

The patient’s medical history included the following: atopic dermatitis, hypothyroidism, osteoporosis, glaucoma, asthma, allergic rhinitis, and breast cancer resulting in a double mastectomy. The patient had been treated for hypothyroidism with levothyroxine, which was continued.

Regarding the family history, the patient’s father had a history of GH deficiency, thyroid cancer, and eczema. The patient’s son and daughter were diagnosed with congenital GH deficiency. The son was 127 cm tall at age 13 with a history of low serum GH levels, required GH therapy to achieve normal height, and was continued on GH therapy into young adulthood because attempted discontinuation led to sleep pattern disruption. The patient’s daughter also had a history of low serum GH levels and achieved a terminal height of 157 cm after undergoing several years of GH therapy through adolescence and into young adulthood.

Regarding social history, the patient adhered to a gluten-free diet, never smoked cigarettes, and denied consuming alcohol- and caffeine-containing beverages. At the time of writing, the patient had retired from her previous job as a guidance counselor and was an instructor for Pilates classes.

At the initial encounter, the patient’s vital signs were within normal limits: heart rate 61 beats per minute, blood pressure 124/64 mmHg, respiratory rate 16 breaths per minute, and oxygen saturation 99% on room air. The patient appeared older than her stated age. The cardiopulmonary examination was noncontributory. The skin was dry over the lateral aspect of the torso and contained erythematous pinpoint lesions without crusting.

**Figure 1:**
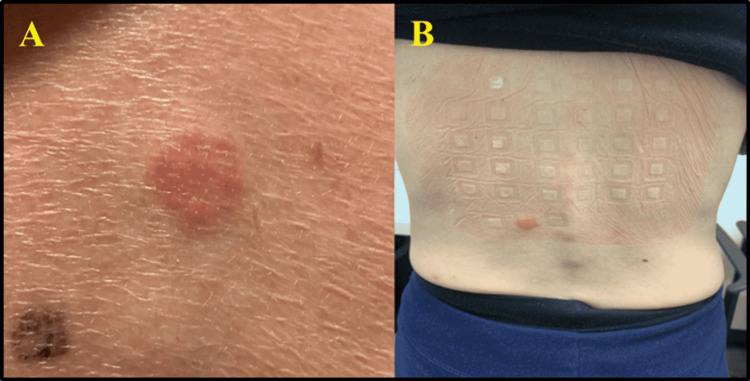
Contact dermatitis following patch testing. A: Contact dermatitis with a positive nickel patch test that did not resolve completely until three weeks after testing was initiated. B: Contact dermatitis with a positive gold patch test that persisted for greater than three months after initiation of patch testing. A positive patch test (Gell-Coombs type 4 delayed hypersensitivity reaction) is a cell-medicated reaction that occurs generally more than 12 hours after allergen exposure and results are usually read 48 to 72 hours after the placement of the patch test.

Skin testing and patch testing were negative for Norditropin Flexpro®. Patch testing was positive for gold (Figure 1), Norditropin, and nickel (Figure 1). The nickel patch test elevation persisted for >three months after administration. A basophil activation test is a flow cytometry test performed after stimuli but is not typically performed in this clinical scenario and would not have been reimbursed by commercial insurance.

**Figure 2:**
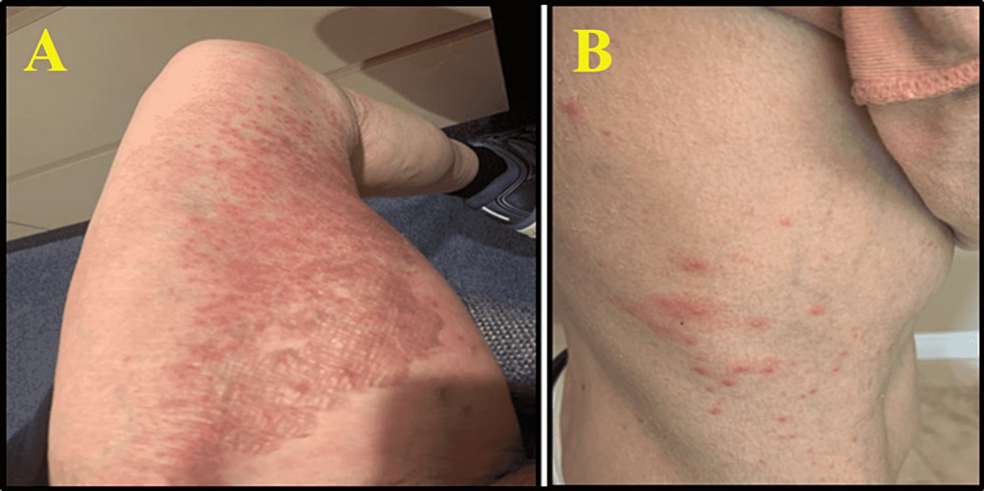
Dermal eruptions in response to growth hormone therapy. A: The severe initial rash that developed steadily on the patient’s left thigh, in addition to a rash on the upper extremities bilaterally (not shown) in response to the administration of 0.425 mg Norditropin Flexpro®. B: After the 0.425 mg dose of Norditropin FlexPro® was discontinued, the patient was placed on 0.1 mg of Norditropin FlexPro® and developed a rash on the torso on day four following Norditropin FlexPro® administration.

The patient was started on 0.425 mg of Norditropin FlexPro® to treat GH deficiency. One year after the diagnosis of adult GH deficiency was established and replacement with GH was initiated, the patient developed nonspecific chronic urticaria, carpal tunnel syndrome, and headaches. The glucagon stimulation testing activated GH appropriately and suggested normal levels of GH with Norditropin Flexpro®. The pruritic rash (Figure 2, Panel A) started as erythematous papules coalescing into plaques on the trunk that eventually spread to the axillae and inner thighs with the erosion of the epidermis that resembled drug exanthems without mucous membrane involvement but with Nikolsky sign positivity. There was no fever.

**Figure 3:**
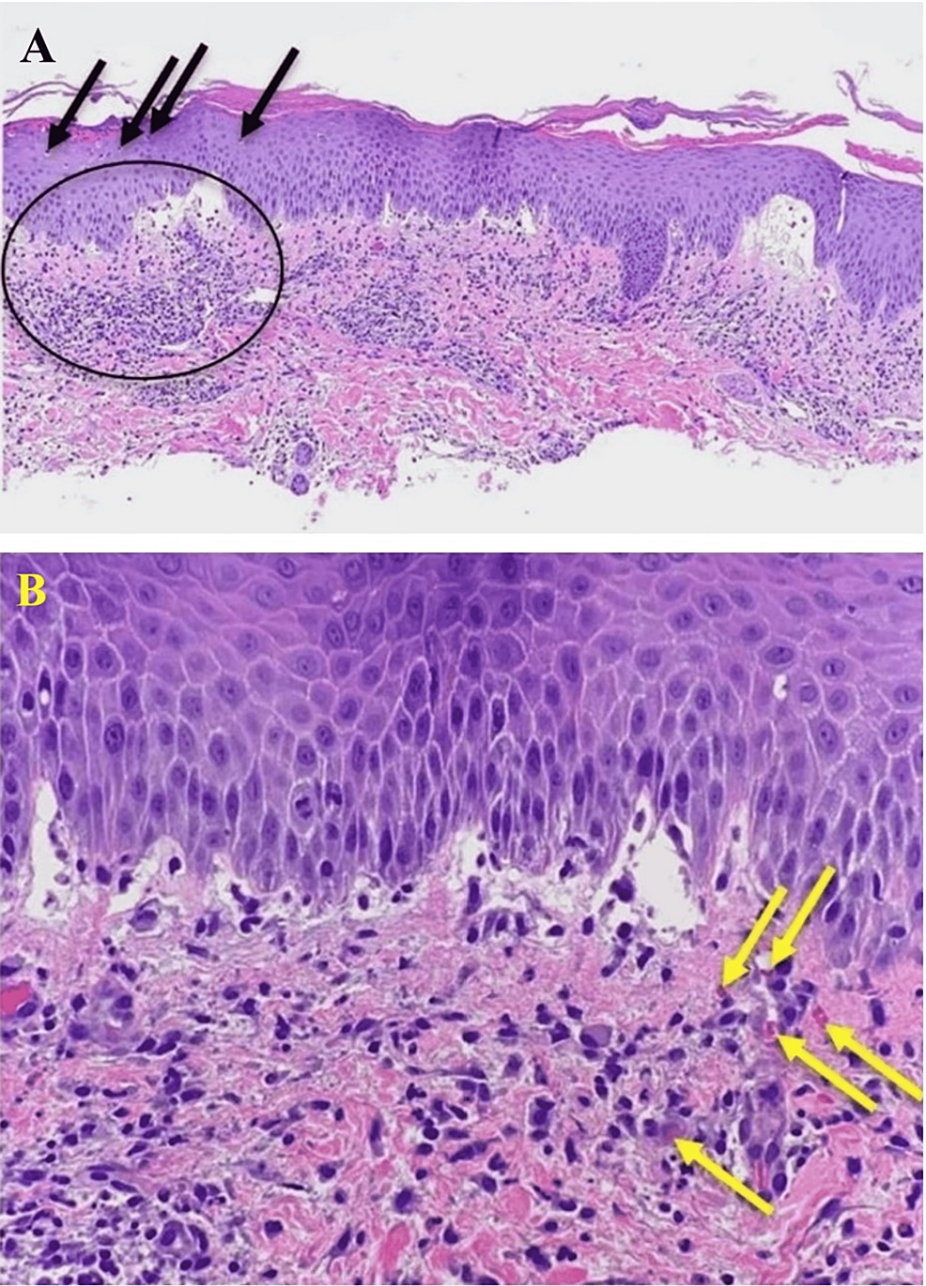
Skin biopsy results were consistent with symmetric drug-related intertriginous/flexural eruption. A: Hematoxylin and eosin stain of epidermis at 100× magnification showing spongiotic dermatitis (black circle) associated with parakeratosis, neutrophilic infiltration of the stratum corneum (black arrows), as well as subepidermal edema. B: Spongiotic dermatitis and subepidermal edema with mixed inflammatory cell infiltrates that include eosinophils found within the dermis (yellow arrows).

The rash was biopsied, and the patient was presented at a University Dermatology Grand Rounds [10] where the panel of dermatologists concluded the rash to be most consistent with symmetric drug-related intertriginous and flexural exanthema (Figure 3).

The patient was instructed to discontinue Norditropin FlexPro®; the rash resolved spontaneously over several months. GH and IGF-1 levels during these months were measured and were concurrently low. GH was negligible at 0.2 ng/mL (reference range: ≤7.1 ng/mL), and IGF-1 had a lower normal baseline of 82 mg/mL (reference range: 41-273 mg/mL). The deficient GH level was again confirmed by an abnormal glucagon stimulation testing (i.e., insufficient GH release).

Subsequent to glucagon stimulation testing, Norditropin FlexPro® was restarted at a dosage of 0.1 mg, causing the patient to develop a milder version of the rash four days after the first injection (Figure 2B). The medication was stopped, but GH levels were still negligible whereas IGF-1 remained at a low normal baseline of 89 mg/mL (reference range: 41-273 mg/mL). The patient was not treated with any other medication for GH deficiency. However, prior to a graded dose challenge with Norditropin®, the patient did previously receive omalizumab for the treatment of chronic urticaria. For treatment of her unrelated urticaria diagnosis, the patient had previously received two doses of omalizumab (anti-immunoglobulin E [IgE]) of 150 mg each with the last dose three days prior to the graded dose challenge. The omalizumab was a treatment for the patient’s urticaria. The omalizumab did address the patient’s skin hives but, as anticipated, did not prevent her delayed allergic rash from Norditropin. The patient’s allergic reaction to Norditropin was delayed hypersensitivity rash which is cell-mediated and not IgE-mediated. Therefore, the administration of the anti-IgE omalizumab as expected did not prevent the patient’s Norditropin reaction or skin response.

The patient was registered as direct admission to the emergency room at the University Hospital for a rapid antigen coronavirus disease 2019 (COVID-19) testing after having received two COVID-19 vaccinations and re-testing four months after vaccination. She was then directly admitted to a non-COVID-19 intensive care unit with direct bedside supervision by a registered nurse and a physician board certified in internal medicine, allergy/immunology, and pulmonary diseases. The patient brought a Norditropin® pen which our pharmacy team attached to a compatible syringe for dilutions. A graded dose challenge at a final dosage of 0.1 mL was performed and the patient was monitored for allergic and other adverse drug reactions, which did not occur. At the time of writing, the patient has been maintained on Norditropin Flexpro® 0.1 mL and has not experienced any adverse reactions, including recurrent skin eruptions.

## Discussion

A graded dose challenge is similar to a desensitization protocol. Possible mechanisms of a graded dose challenge are alteration in expression of antigen surface receptors, generation of IgG blocking antibodies, altered cellular signaling in basophils and mast cells, and/or increased expression of inhibitory receptors and phosphatases [11]. Clinically, a graded dose challenge involves gradual administration of a medication to a patient who has a history of dermatologic reaction(s) to the medication being administered; however, the reactions are not IgE or allergy-mediated [12]. A successful graded dose challenge has been reported in the literature in which Humatrope®, a somatotropin analog other than that used in the case presented here (i.e., Norditropin®), was administered to a child [9]. Drug desensitization is used to allow the administration of the medication to allergic individuals. Frequently, drug desensitization is used for IgE-mediated drug allergic reactions. However, drug desensitization can also be used for non-IgE-mediated reactions.

Table 2 outlines a protocol for increased dosing, if indicated, for Humatrope® dosing in the pediatric population. Briefly, this protocol entails the utilization of three bags of varying concentrations diluted to yield a series of increasing doses of Humatrope® that may be successively administered. Briefly, bag #1 would contain 0.005 mg in 100 mL normal saline (concentration 5.00 × 10^-5^ mg/mL); bag #2 would contain 0.5 mg in 100 mL normal saline (concentration 5.00 × 10^-3^ mg/mL); and bag #3 would contain 2.5 mg in 50 mL normal saline (concentration 5.00 × 10^-2^ mg/mL).

**Table 2:** Humatrope® recombinant human growth hormone intravenous desensitization protocol.

Bag#	Step	Concentration (mg/mL)	Alaris pump rate (mL/hour)	Volume (mL per 15 minutes)	Dose (mg)	Cumulative dose (mg)
1	1	5.00 × 10^-5^	0.4	0.1	5.00 × 10^-6^	5.00 × 10^-6^
1	2	5.00 × 10^-5^	0.8	0.2	1.00 × 10^-5^	1.50 × 10^-5^
1	3	5.00 × 10^-5^	1.6	0.4	2.00 × 10^-5^	3.50 × 10^-5^
1	4	5.00 × 10^-5^	3.2	0.8	4.00 × 10^-5^	7.50 × 10^-5^
1	5	5.00 × 10^-5^	4	1	5.00 × 10^-5^	1.25 × 10^-4^
1	6	5.00 × 10^-5^	8	2	1.00 × 10^-4^	2.25 × 10^-4^
1	7	5.00 × 10^-5^	16	4	2.00 × 10^-4^	4.25 × 10^-4^
1	8	5.00 × 10^-5^	32	8	4.00 × 10^-4^	8.25 × 10^-4^
2	9	5.00 × 10^-3^	0.4	0.1	5.00 × 10^-4^	1.33 × 10^-3^
2	10	5.00 × 10^-3^	0.8	0.2	1.00 × 10^-3^	2.33 × 10^-3^
2	11	5.00 × 10^-3^	1.6	0.4	2.00 × 10^-3^	4.33 × 10^-3^
2	12	5.00 × 10^-3^	3.2	0.8	4.00 × 10^-3^	8.33 × 10^-3^
2	13	5.00 × 10^-3^	4	1	5.00 × 10^-3^	1.33 × 10^-2^
2	14	5.00 × 10^-3^	8	2	1.00 × 10^-2^	2.33 × 10^-2^
2	15	5.00 × 10^-3^	16	4	2.00 × 10^-2^	4.33 × 10^-2^
2	16	5.00 × 10^-3^	32	8	4.00 × 10^-2^	8.33 × 10^-2^
3	17	5.00 × 10^-2^	4	1	5.00 × 10^-2^	1.33 × 10^-1^
3	18	5.00 × 10^-2^	8	2	1.00 × 10^-1^	2.33 × 10^-1^
3	19	5.00 × 10^-2^	16	4	2.00 × 10^-1^	4.33 × 10^-1^
3	20	5.00 × 10^-2^	32	8	4.00 × 10^-1^	8.33 × 10^-1^
	21	Undiluted		0.1 (SQ)	5.00 × 10^-1^	1.33
	22	Undiluted		0.4 (SQ)	2.00	3.33

In patients who have a history of receiving chemotherapy, the likelihood of GH deficiency from hypopituitarism is increased [13]. In the past, the patient underwent a double mastectomy for breast cancer but did not receive chemotherapy. The rash was temporally related to exposure to Norditropin FlexPro®.

Patients with a history of atopy are at risk of developing severe and prolonged contact dermatitis [14]. Constant allergen stimulation, age, and atopy may predispose to long-lasting patch test reactions. This could explain why our patient with a history of atopy and severe contact dermatitis developed a severe rash after exposure to Norditropin FlexPro®. Severe hypersensitivity reactions have also been reported in patients treated with Norditropin FlexPro® [15]. Allergic and non-allergic rashes to GH therapy have been reported but they are relatively uncommon [8]. Non-allergic rashes to GH can stimulate an underlying skin disorder to flare up.

This patient presented with a rare form of heritable adult GH deficiency. GH deficiency was present in her two children. The son and daughter are undergoing full gene sequencing for specific genes that can cause isolated GH deficiency. These genes include *GH1*, *BTK*, *GHRHR*, and *RNPC3* [16]. The sensitivity of the patch test is only 32%, so a negative patch test to Norditropin® does not entirely exclude delayed contact dermatitis. Her clinical finding of a rash that developed days after exposure is clinically consistent with delayed contact dermatitis [17].

## Conclusions

The case presented describes a patient who successfully tolerated a graded dose challenge of an adult patient to GH replacement therapy (as Norditropin®) under supervision in an intensive care unit. Previously, there has also been GH replacement therapy via graded dose challenge successfully performed on a child using another formulation of somatotropin (Humatrope®). Hence, this case lends support that graded dose challenge with somatotropin analogs may be considered for patients with isolated GH deficiency such as in the case presented here.
